# Anistropically varying conductivity in irreversible electroporation simulations

**DOI:** 10.1186/s12976-017-0065-6

**Published:** 2017-11-01

**Authors:** Nicholas Labarbera, Corina Drapaca

**Affiliations:** 1 Department of Engineering Science & Mechanics, The Pennsylvania State University, 212 Earth-Engineering Sciences Bldg., University Park, 16802 PA USA; 2 Center for Neural Engineering, The Pennsylvania State University, 409C Earth-Engineering Science Building, University Park, 16802 PA USA

**Keywords:** Irreversible electroporation, Anisotropic conductivity, Tumor ablation, Treatment planning

## Abstract

**Background:**

One recent area of cancer research is irreversible electroporation (IRE). Irreversible electroporation is a minimally invasive procedure where needle electrodes are inserted into the body to ablate tumor cells with electricity. The aim of this paper is to propose a mathematical model that incorporates a tissue’s conductivity increasing more in the direction of the electrical field as this has been shown to occur in experiments.

**Method:**

It was necessary to mathematically derive a valid form of the conductivity tensor such that it is dependent on the electrical field direction and can be easily implemented into numerical software. The derivation of a conductivity tensor that can take arbitrary functions for the conductivity in the directions tangent and normal to the electrical field is the main contribution of this paper. Numerical simulations were performed for isotropic-varying and anisotropic-varying conductivities to evaluate the importance of including the electrical field’s direction in the formulation for conductivity.

**Results:**

By starting from previously published experimental results, this paper derived a general formulation for an anistropic-varying tensor for implementation into irreversible electroporation modeling software. The anistropic-varying tensor formulation allows the conductivity to take into consideration both electrical field direction and magnitude, as opposed to previous published works that only took into account electrical field magnitude.

The anisotropic formulation predicts roughly a five percent decrease in ablation size for the monopolar simulation and approximately a ten percent decrease in ablation size for the bipolar simulations. This is a positive result as previously reported results found the isotropic formulation to overpredict ablation size for both monopolar and bipolar simulations. Furthermore, it was also reported that the isotropic formulation overpredicts the ablation size more for the bipolar case than the monopolar case. Thus, our results are following the experimental trend by having a larger percentage change in volume for the bipolar case than the monopolar case.

**Conclusions:**

The predicted volume of ablated cells decreased, and could be a possible explanation for the slight over-prediction seen by isotropic-varying formulations.

## Background

Electroporation is an electrically-driven biological process that uses externally applied electric fields to briefly open the nano-sized pores in a cell’s membrane. On one hand, electroporation can be used to transport drugs and genetic materials into cells [1]. On the other hand, a strong enough electrical field can irreversibly damage the cell’s membrane and cause cell death. This process is called irreversible electroproation (IRE), and has gained interest as a possible tumor ablation method [2].

A cell’s viability is directly related to the electrical field it is exposed to. Each cell is characterized by a critical value so that when the cell is exposed to an electrical field above this critical value it loses its ability to reseal the pores in its membrane and eventually dies. Any electrical field below this critical value, either does not form pores or the pores close when the electrical field is removed. The critical value depends on various factors such as tissue type, tumor stage, electrode configuration and size, and the direction of the applied electric field. The success of an IRE-based therapy for tumors relies on this critical value and thus knowledge about the optimal configuration and size of the electrodes as well as the applied voltage needed to completely ablate the tumor while minimizing the damage to healthy tissue is relevant to clinicians.

Mathematical models of electroporation and corresponding numerical simulations able to predict the electric field distribution in a tissue of interest for various geometries and positions of electrodes could play an important, complementary role in the design of individual treatment protocols [3, 4]. In particular, it is well established in the literature that electroporation can affect the conductivity of tissue [5, 6]. The large increase in conductivity is a result of the membrane’s pores opening during electroporation [7]. Corovic et al. [8] recommended to incorporate in mathematical models a conductivity that is dependent on the electrical field strength to increase accuracy. Experiments performed by Mezeme et al. suggest that electroporation does not always isotropically increase the conductivity of the tissue, but conductivity is increased more in the direction tangent to the electric field [9]. Their experiments determined the conductivity of electroporated chicken livers by using magentic resonance electrical impedance tomography. The result of an anistropic conductivity tensor can be explained by the pores forming in the cell membrane with a bias towards the direction of the electrical field [10]. This is because the voltage drop across the cell membrane is not equal around the entire cell, but is largest where the cell membrane is perpendicular to electrical field [10].

Therefore, in this paper we propose a mathematical model that incorporates a tissue’s conductivity increasing more in the direction of the electrical field. To do so, it was required for us to derive a formulation for the conductivity tensor such that conductivity increases by different amounts in the direction tangent and normal to the electrical field and can be easily implemented in numerical methods such as finite elements. Numerical simulations were performed for isotropic and anisotropic varying conductivities to evaluate the importance of including the electrical field’s direction in the formulation for conductivity.

## Method

This section will discuss the details of the mathematical models used for comparing the well established isotropically increasing-conductivity formulation and this paper’s anistropically increasing-conductivity formulation. The simulations used for comparison will be based off the irreversible electroporation experiments and simulations performed by Castellvi et al. [11]. This work will use their experimentally determined parameters to compare an isotropic formulation to an anisotropic formulation.

### Governing equations

In numerical simulations for treatment planning, it is assumed that the applied direct current electric field is at steady state [12]. The electrical potential, *U*, satisfies the differential form of Gauss’s law at steady state:

1 $$ \nabla \cdot (\sigma \nabla U) = 0  $$

where *σ* is the tissue’s conductivity. Then the electric field *E* is given by: *E*=−∇*U*. If *σ* is a constant, then Eq. (1) becomes the well-known Laplace equation. However, the conductivity of tissue is not in general constant but increases during electroporation [6], and therefore, in this paper, we model the conductivity as a function of the electric field, *σ*=*σ*(*E*).

We model the isotropic varying conductivity as a sigmoid Gompertz curve [8, 13, 14]:

2 $$ \sigma(\|E\|) = \sigma_{0} + (\sigma_{max} - \sigma_{0}) \cdot \exp [ -A \cdot \exp(-B \cdot \|E\|) ]  $$

where *A* and *B* are unitless coefficients, *σ*_0_ is the the base conductivity before electroporation, *σ*_*max*_ is the maximum conductivity a tissue can achieve after electroporation, and ∥*E*∥ is the *l* ^2^− norm of the electrical field. By definition, the *l* ^2^− norm of the electrical field is $\|E \| = \sqrt {E_{x}^{2} + E_{y}^{2} + E_{z}^{2} }$ where *E*_*x*_, *E*_*y*_, *E*_*z*_ is the *x*, *y*, *z* component of the electrical field respectively. The values of *A* and *B* are found by fitting the curve for conductivity to experimental data.

If the conductivity is isotropic, then *σ* is a scalar. However, the conductivity is anisotropic when the conductivity increases more in the direction of the electrical field. This results in the conductivity, *σ*, being represented by a rank-2 tensor.

We will compare two different cases. The first being when *σ* does not take into account the direction of the electrical field (isotropic-varying) and when *σ* does take into account the direction of the electrical field (anisotropic-varying). Both cases will solve Eq. 1; the only difference will be in how *σ* is defined.

### Isotropic-varying formulation

This is the well established model that is often used for IRE simulation predictions. It will be used for comparison with our anisotropic-varying formulation. The isotropic-varying formulation has the conductivity increase equally in all directions. Hence, it being refereed to as the isotropic formulation in this paper. The advantage of using an isotropic formulation is that the conductivity is represented by a single scalar, and the direction of the electrical field does not need to be considered. It is also less computationally intensive.

In the isotropic formulation, the changes in conductivity from electroporation can be taken into account with a sigmoid Gompertz curve for the conductivity [8, 14, 15]. The same expression for conductivity as was found in [11] which is

3 $$ \sigma(\| E\|) = 0.03 + 0.35 * \text{exp} (- \text{exp} (-0.01 (\| E \| - 250))  $$

will be used for the isotropic formulation.

### Anisotropic-varying tensor derivation

In this paper, we propose a conductivity that takes into account the electrical field’s direction and magnitude. We wish to formulate the conductivity tensor such that the conductivity increases more in the direction of the electrical field. Since the electrical field does not increase equally in all directions, we will refer to this formulation as the anisotropic formulation in this paper.

We now wish to derive the matrix representation for the conductivity tensor for the anisotropic-varying case such that it can be implemented into a numerical scheme. This is the main contribution of the paper.

We begin by assuming the conductivity tensor is a real valued symmetric matrix. This is common assumption and is justified by assuming conductivity is independent of current flow being positive or negative. All symmetric matrices are diagnolizable. Therefore, the conductivity tensor is diagonalizable.

The anisotropic case can have a different conductivity in the direction of the electrical field than perpendicular to it. If we make the assumption that the conductivity in the direction of the electrical field is always equal or greater than any other direction, and that the conductivity in a direction perpendicular to the electrical field is always equal or less than any non-normal direction then the directions tangent and normal to the electrical field are principal directions. These assumptions are justified according to the experimental work found in [9].

Let *σ*_*t*_ be an arbitrary function representing the conductivity in the direction tangent to the electrical field. Also let *σ*_*n*_ and *σ*_*b*_ be arbitrary functions representing conductivity in the direction normal and bi-normal to the electrical field respectively. By treating *σ*_*t*_, *σ*_*n*_, and *σ*_*b*_ as arbitrary functions, we are providing a derivation that is valid for any expression for *σ*_*t*_, *σ*_*n*_, and *σ*_*b*_ which may someday be ascertained through experiments.

A tensor is diagonal when the principal directions are chosen as the basis representation. We will therefore consider a coordinate system comprised of a unit vector tangent to the electrical field and two unit vectors perpendicular to the electrical field. A coordinate system consisting of a tangent vector, normal vector, and bi-normal vector, is sometimes referred to as a Frenet-Serret frame. These three vectors form an orthonormal basis in which the basis vectors point in the principal directions of the conductivity. Since the Frenet-Serret basis vectors are aligned with the principal directions then the conductivity tensor matrix representation is diagonal in the Frenet-Serret coordinate system and can be represented as

4 $$ \boldsymbol{\sigma}_{f} = \left(\begin{array}{ccc} \sigma_{t} & 0 & 0 \\ 0 & \sigma_{n} & 0 \\ 0 & 0 & \sigma_{b}\\ \end{array} \right)  $$

where ***σ***_*f*_ is the matrix representation of the conductivity tensor in the Frenet-Serret coordinate system. It will be assumed that the conductivity is the same for any direction normal to the electrical field. This implies that both the normal and bi-normal direction conductivities are equal,

5 $$ \sigma_{b} = \sigma_{n}.  $$

Equation 5 simplifies Eq. 4 to

6 $$ \boldsymbol{\sigma}_{f} =\left(\begin{array}{ccc} \sigma_{t} & 0 & 0 \\ 0 & \sigma_{n} & 0 \\ 0 & 0 & \sigma_{n} \\ \end{array} \right).  $$

where ***σ***_*f*_ represents a linear transformation that takes a vector from the Frenet-Serret frame and outputs a vector in the Frenet-Serret frame. This can be restated as

7 $$ \boldsymbol{\sigma}_{f} : \mathfrak{R}^{3}_{f} \rightarrow \mathfrak{R}^{3}_{f}  $$

where $\mathfrak {R}_{f}^{3}$ is the space of three dimensional vectors represented by the Frenet-Serret basis. However, for calculations, we would like to have the conductivity tensor in the Cartesian coordinate system as it is often impractical to implement the Frenet-Serret matrix representation into a numerical scheme such as finite elements. Thus, we wish to derive

8 $$ \boldsymbol{\sigma}_{c} : \mathfrak{R}^{3}_{c} \rightarrow \mathfrak{R}^{3}_{c}  $$

where ***σ***_*c*_ is the matrix representation of the conductivity tensor in the Cartesian coordinate system, and $\mathfrak {R}^{3}_{c}$ is the space of three dimensional vectors represented by the standard Cartesian basis. We will use the known form of the matrix representation of the conductivity tensor in the Frenet-Serret frame to derive the matrix representation of the conductivity tensor in the Cartesian frame.

Since the Frenet-Serret frame and the Cartesian coordinate system are both orthonormal, a change of basis between the two can be represented by a rotation through an angle *θ* about the z-axis and a rotation *ϕ* about the y-axis. Note only two rotations instead of three are necessary because the conductivity has been assumed to be equal in all normal directions.

The rotation tensor, ***R***(*θ*,*ϕ*), is a linear transformation such that

9 $$ \boldsymbol{R}: \mathfrak{R}^{3}_{c} \rightarrow \mathfrak{R}^{3}_{f}  $$

**Fig. 1:**
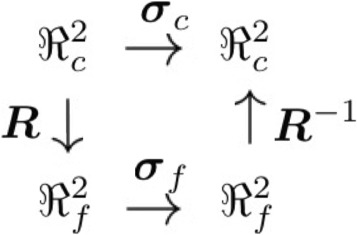
Function Diagram for the conductivity tensor

A function diagram outlining the relationship between ***σ***_*c*_, ***σ***_*f*_, and **R** can be found in Fig. 1.

We can decompose ***R*** into two subsequent rotations. The first rotation will be about the z-axis, ***R***_*z*_(*θ*), and can be expressed by the matrix,

10 $$ \boldsymbol{R}_{z}(\theta) = \left(\begin{array}{ccc} \cos (\theta) & -\sin (\theta) & 0 \\ \sin (\theta) & \cos (\theta) & 0 \\ 0 & 0 & 1 \\ \end{array} \right)  $$

where the angle *θ* is obtained through the relationship

11 $$ \theta = \tan^{-1} \left(\frac{E_{y}}{E_{x}}\right).  $$

The quantities *E*_*x*_ and *E*_*y*_ represent the x and y components of the electrical field respectively. Similarly, the second rotation will be about the y-axis, ***R***_*y*_(*ϕ*), and is expressed by the matrix,

12 $$ \boldsymbol{R}_{y}(\phi) = \left(\begin{array}{ccc} \cos (\phi) & 0 & -\sin (\phi) \\ 0 & 1 & 0 \\ \sin (\phi) & 0 & \cos (\phi) \\ \end{array} \right)  $$

where *ϕ* is the angle described by the function

13 $$ \phi = \tan^{-1} \left(\frac{E_{z}}{E_{x}}\right).  $$

The quantity *E*_*z*_ represents the z component of the electrical field. The rotation tensor can be then be expressed as

14 $$ \boldsymbol{R}(\theta,\phi) = \boldsymbol{R}_{y}(\phi) \boldsymbol{R}_{z}(\theta)  $$

Using properties of rotation matrices, ***R*** ^−1^ is

15 $$ \boldsymbol{R}^{-1} = (\boldsymbol{R}_{y}(\phi) \boldsymbol{R}_{z}(\theta))^{-1} = \boldsymbol{R}_{z}(-\theta) \boldsymbol{R}_{y}(-\phi)  $$

We are now able to express the conductivity tensor in matrix form with the domain and codomain being the Cartesian frame. This is accomplished by applying the rotation matrices in the following order:

16 $$ \boldsymbol{\sigma}_{c} = \boldsymbol{R}^{-1}\boldsymbol{\sigma}_{f} \boldsymbol{R}  $$

17 $$ \boldsymbol{\sigma}_{c} = \boldsymbol{R}_{z}(-\theta) \boldsymbol{R}_{y}(-\phi) \boldsymbol{\sigma}_{f} \boldsymbol{R}_{y}(\theta) \boldsymbol{R}_{z}(\phi)  $$

18 $$ \boldsymbol{\sigma}_{c} = \left(\begin{array}{ccc} \sigma_{11} & \sigma_{12} & \sigma_{13} \\ \sigma_{12} & \sigma_{22} & \sigma_{23} \\ \sigma_{13} & \sigma_{23} & \sigma_{33} \end{array}\right)  $$

where

19 $$ \sigma_{11} = \cos^{2}(\theta) \left(\sigma_{n} \sin^{2}(\phi)+\sigma_{t} \cos^{2}(\phi)\right)+\sigma_{n} \sin^{2}(\theta)  $$

20 $$ \sigma_{22} = \sin^{2}(\theta) \left(\sigma_{n} \sin^{2}(\phi)+\sigma_{t} \cos^{2}(\phi)\right)+\sigma_{n} \cos^{2}(\theta)  $$

21 $$ \sigma_{33} = \sigma_{n} \cos^{2}(\phi)+\sigma_{t} \sin^{2}(\phi)  $$

22 $$ \sigma_{12} = \sin (\theta) \cos (\theta) (\sigma_{n}-\sigma_{t}) \cos^{2}(\phi)  $$

23 $$ \sigma_{13} = \cos(\theta) (\sigma_{n}-\sigma_{t}) \sin(\phi) \cos(\phi)  $$

24 $$ \sigma_{23} = \sin(\theta) (\sigma_{n}-\sigma_{t}) \sin(\phi) \cos(\phi)  $$

The form of an anisotropic varying conductivity tensor has been derived with arbitrary functions for the conductivity in the tangent and normal directions. Therefore, this formulation is valid for any functions of conductivity for *σ*_*t*_ and *σ*_*n*_, including those dependent on electrical field strength.

For the anisotropic case simulations, the conductivity used for the tangent direction, *σ*_*t*_, will have the same form as the conductivity for the isotropic case:

25 $$ \sigma_{t} (\| E \|) = \sigma (\| E \|) =0.03 + 0.35 * \exp (- \exp (-0.01 (\| E \| - 250))  $$

This was chosen because the measurements used to experimentally determine the form of the conductivity is often done using electrodes that are aligned with the electrical field.

As shown in [9], the conductivity tensor becomes more anisotropic as the electrical field strength increases. This ratio will be modelled using a sigmoid function, *σ*_*Δ*_, where

26 $$ \sigma_{\Delta} (\| E \|) = 0.30 * \exp (-\exp (-0.01*(\| E\|-250)))  $$

is an adaptation of the data obtained in the experiments reported in [9]. Due to the limited amount of experimental data, this was the authors’ best guess as to the form for *σ*_*Δ*_. But since the derivation was done in a general setting, it will be possible to plug in different formulations at a later time as they become available from experimental data. The approximation can be combined with the previous expression for *σ*_*t*_ to obtain the following expression for the conductivity in the perpendicular direction:

27 $$ \sigma_{n} = (1- \sigma_{\Delta}) \sigma_{t}.  $$

It is worth noting, that our form for *σ*_*Δ*_ results in *σ*_*t*_ and *σ*_*n*_ being equal until the onset of electroporation. In other words, the conductivity is isotropic until the onset of electroporation. During electroproation, the conductivity tensor becomes more anisotropic with increasing electrical fields up until a value of *σ*_*t*_ being 30% larger than *σ*_*n*_.

### Boundary conditions

Boundary conditions need to be specified before Eq. 1 can be solved. It is common for boundaries of the electrode to be Dirchlet type and the remaining boundaries to be Neumann type [16]. Specifically, the boundary condition for a charged electrode will be

28 $$ U = V_{0}  $$

where *V*_0_ is the applied voltage of the electrode. For a grounded electrode, *V*_0_ would be zero and the boundary condition would read

29 $$ U = 0.  $$

The boundaries not in contact with an electrode, are often modeled as electrically insulating,

30 $$ \frac{\partial U}{\partial n} = 0  $$

where *n* is the outward pointing normal [16].

### Monopolar geometry

**Fig. 2:**
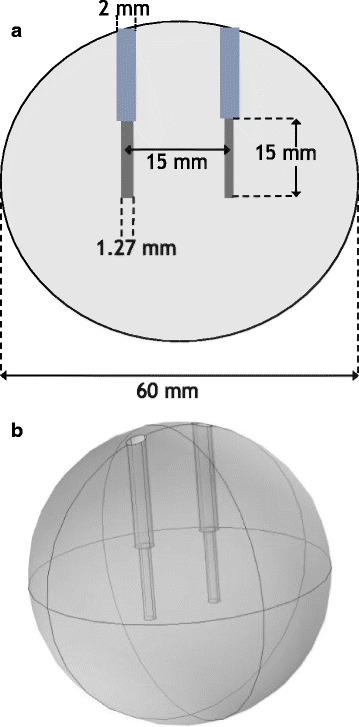
Schematic of the simulations performed by Castellvi and used for the monopolar test case

The geometry used for the monopolar simulations was the same as used by Castellvi et al. It consisted of two monopolar electrodes spaced 15 mm apart embedded in a 60 mm sphere. A schematic of the simulation geometry is shown in Fig. 2. The outer surface and electrode sleeves were modelled as electrically insulating. That is to say, on those boundaries the condition

31 $$ \frac{\partial U}{\partial n} = 0  $$

where *n* is the outward pointing normal is enforced. The remaining boundary conditions is to set the active area of one electrode to ground by enforcing the condition

32 $$ U= 0,  $$

and to enforce the condition that the active area of the other electrode is held at a constant voltage by enforcing the equation

33 $$ U= V_{0}.  $$

### Bipolar geometry

**Fig. 3:**
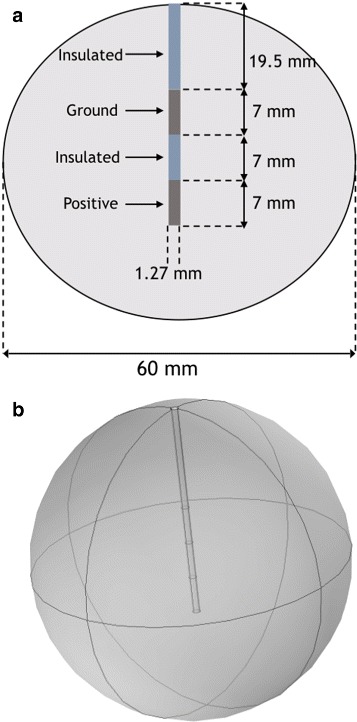
Schematic for the bipolar test case

The domain used for the bipolar simulations was a 60 mm sphere. The electrode has a diameter of 1.5 mm and a length of 40.5 mm. The last 7 mm of the electrode are set to the positive voltage. The next 7 mm of the electrode is insulated, and then followed by 7 mm of the electrode set to ground. The remaining portion of the electrode is set to an insulating boundary condition as is the outer sphere. A schematic of the domain is shown in Fig. 3.

### Finite element solver

All simulations were performed using the commercial finite element software COMSOL. The meshes used for the simulations consisted of approximately 500,000 cells, and were run using 2.2 GHz Intel Xeon processors.

## Results

### Monopolar simulations

**Fig. 4:**
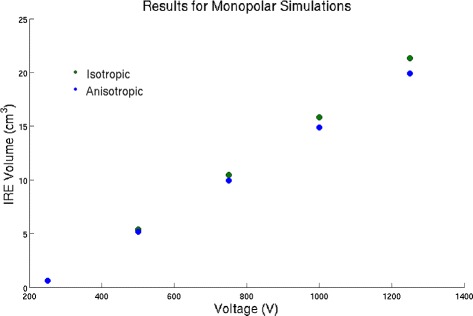
Ablation volumes for the two different formulations with the monopolar geometry

Both formulations were run with various voltages. A comparison of the the ablation volume can be found in Fig. 4. It can be seen that the anistropoic formulation predicts a slightly smaller ablation zone than the isotropic formulation. For all simulations, a volume was considered irreversibly electroporated if the magnitude of the electrical field was 184 V/cm or greater. This value was determined experimentally in [11] for the cases this work recreated for comparison.

The shape between the anisotropic and the isotropic formulation are similar except for the anisotropic formulation resulting in a slightly less spherical ablation zone than the isotropic formulation. Similar results were seen for other voltages.

### Bipolar simulations

**Fig. 5:**
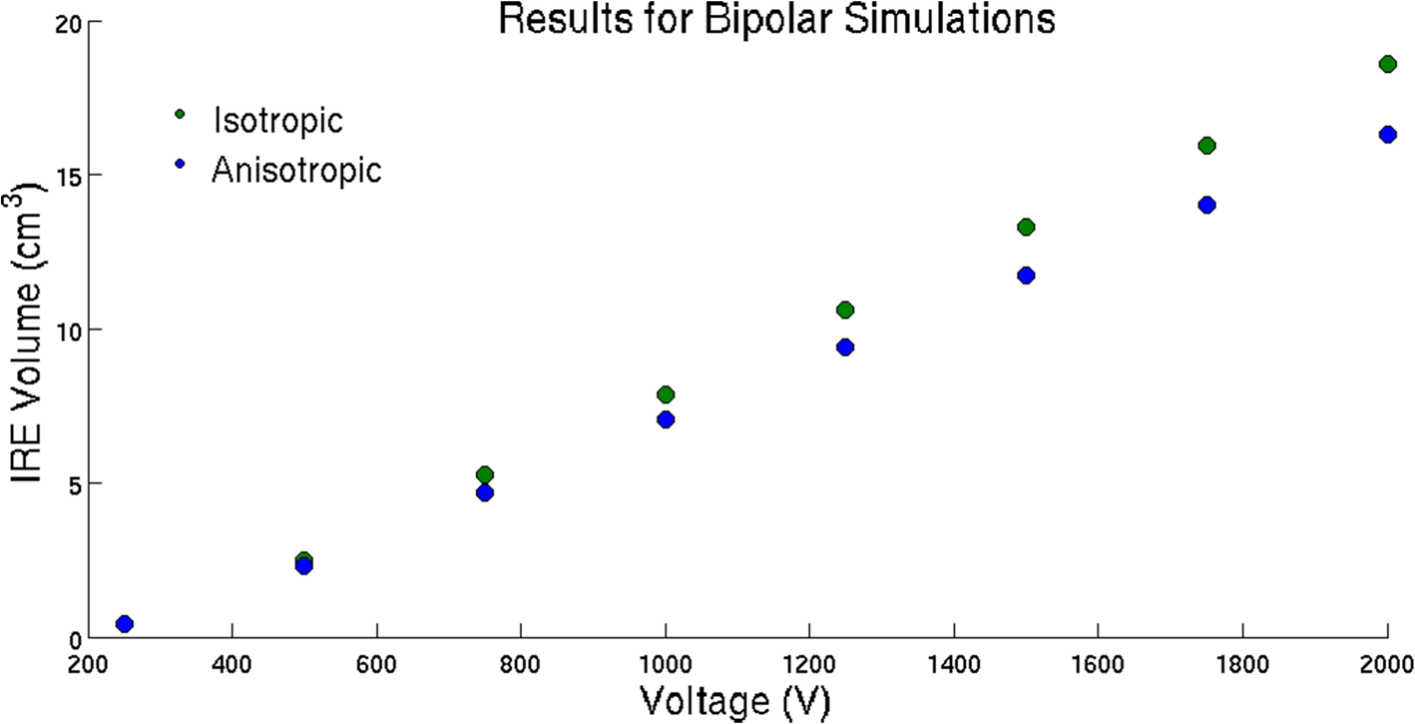
Ablation volumes for the two different formulations with the bipolar geometry. No experimental data was available

For the bipolar geometry, the volume predicted to be ablated is much lower for the anisotropic simulations than for the isotropic simulations. A summary of the results are displayed in Fig. 5. Notice that there is a larger percentage change between the isotropic and the anisotropic cases for the bipolar probe than for the monopolar probes. Ablation zones for the bipolar geometry are displayed in Figs. 5 for an applied voltage of 750 V. This was done to show that the anistotropic-varying formulation produces an ablation shape that is qualitatively similar, but quantitatively slightly smaller. Similar ablation shapes were seen for the other voltages as well.

## Discussion and conclusions

This paper derived a general formulation for an anistropic-varying tensor for implementation into irreversible electroporation modeling software. The anistropic-varying tensor formulation allows the conductivity to take into consideration both electrical field direction and magnitude, as opposed to previous published works that only took into account electrical field magnitude. It was derived for arbitrary functions for *σ*_*t*_ and *σ*_*n*_, and is therefore applicable irregardless of the form chosen for *σ*_*t*_ and *σ*_*n*_. The formulation was compared to more commonly used isotropically varying formulations, and was found to decrease the predicted ablation zone for both monopolar and bipolar electrode setups.

Further experimental and numerical work is necessary before any definitive conclusions can be drawn on the importance of including an anisotropic-varying formulation in ablation zone predictions. The hope of this work is to encourage further research on how electrical fields can affect the conductivity tensor in different directions because more experimental data is necessary before any definitive conclusions can be drawn.

## Abbreviations

IRE: Irreversible electroporation
UQ: Uncertainty quantification
